# Multiparametric MRI radiomics for noninvasive risk stratification of pediatric neuroblastoma: a pilot study

**DOI:** 10.1038/s41598-026-65580-8

**Published:** 2026-08-10

**Authors:** M. S. Anders, F. Mollica, T. Meyer, R. Tahan, H. E. Deubzer, S. Veldhoen, C. Metz

**Affiliations:** 1 Charité – Universitätsmedizin Berlin, Corporate Member of Freie Universität Berlin and Humboldt-Universität zu Berlin, Pediatric Radiology, Berlin, Germany; 2 Charité – Universitätsmedizin Berlin, Corporate Member of Freie Universität Berlin and Humboldt-Universität zu Berlin, Radiology, Berlin, Germany; 3 Charité – Universitätsmedizin Berlin, Corporate Member of Freie Universität Berlin and Humboldt-Universität Zu Berlin, Pediatric Hematology and Oncology, Berlin, Germany

**Keywords:** Machine learning, Magnetic resonance imaging, Neuroblastoma, Pediatric oncology, Radiomics, Risk stratification, Biomarkers, Cancer, Medical research, Oncology

## Abstract

Neuroblastoma is the most common extracranial solid tumor in children, with risk stratification guiding therapy and prognosis. Although current risk stratification incorporates imaging-based staging, definitive risk assignment still relies on tissue and molecular characterization, highlighting the need for complementary noninvasive imaging biomarkers. The objectives were to evaluate the classification performance of multiparametric magnetic resonance imaging (MRI) radiomics for risk stratification of pediatric neuroblastoma and to determine how MRI sequences, feature-selection methods, and machine learning classifiers influence classification performance. This retrospective single-center feasibility study included 30 children with histologically confirmed neuroblastoma who underwent pre-treatment T1-, T2-, and diffusion-weighted MRI. From each sequence, 208 radiomic features were extracted from the whole-tumor volume and reduced using six feature-selection methods. Principal components of selected features trained six machine learning classifiers. Performance was assessed using a nested leave-one-out cross-validation framework, with predictions aggregated to one per patient before computing performance metrics, for binary classification of low/intermediate-risk versus high-risk neuroblastoma, using clinical risk classification as the reference standard, with pairwise differences evaluated by DeLong test and Benjamini–Hochberg correction. Among 30 children (mean age ± SD: 38 ± 40 months), the highest discrimination between risk groups was achieved using T2-weighted features and the combined T1w + T2w + ADC features, both with XGB (AUC = 0.88 ± 0.06 and 0.88 ± 0.07, respectively); however, the limited sample size prohibited the detection of significant differences between classifiers after correction for multiple comparisons. Features derived from T2-weighted and diffusion-weighted MRI contributed most to accurate classification. The chi-square feature selection method most frequently contributed to high-performing model configurations (30.8%). Multiparametric MRI radiomics based on whole-tumor volumes showed preliminary evidence of feasibility for noninvasive risk stratification of pediatric neuroblastoma, supporting its potential as a complementary imaging biomarker.

## Introduction

Neuroblastoma (NB) is a clinically heterogeneous embryonal malignancy and the most common extracranial solid tumor in children, with a global incidence of ~ 0.28 per 100,000, originating from immature neural-crest–derived sympathetic progenitors. It accounts for 8–10% of pediatric cancers and ~ 15% of childhood cancer-related deaths^1,2^, frequently metastasizing to bone marrow, bone, liver, and lymph nodes^3,4^. Risk stratification into low, intermediate, and high-risk groups guides therapy and predicts outcome^5^, with five-year survival ranging from 90 to 95% in low/intermediate-risk to below 62% in high-risk NB^4,6^.

Magnetic resonance imaging (MRI) is essential for evaluating tumor extent and guiding biopsy, typically using T1-weighted (T1w), T2-weighted (T2w), and diffusion-weighted (DWI) sequences^7–9^. Nevertheless, established risk stratification also requires histopathologic and molecular/genomic information, including histology, MYCN status, chromosomal aberrations, and DNA ploidy^5,10^.

Radiomics extracts quantitative features from medical images, capturing intensity, shape, and texture information^11^. These features characterize tumor heterogeneity and have been linked to histology, proliferation, and genetic alterations^12^. Machine learning classifiers can exploit these high-dimensional features to predict clinical outcomes^13^, though careful dimensionality reduction is needed to avoid overfitting.

Radiomics has been widely investigated in adult cancers such as lung^11^, breast^14^, prostate^15^, brain^16^, and liver^17^. However, applications in pediatric oncology remain limited due to age-related biological differences, smaller cohorts, and imaging variability^18^. In NB, preliminary evidence suggests MRI-based radiomics can predict MYCN amplification and risk groups, although such prior work has typically relied on single-sequence or region-of-interest-based analyses rather than whole-tumor, multiparametric assessment^19^.

This study builds on the current literature by combining whole-tumor 3D segmentation, multiparametric MRI, and a systematic comparison of multiple feature-selection and classifier strategies, aiming to identify models for noninvasive NB risk stratification and addressing a critical gap in early, image-based risk prediction.

## Materials and methods

### Institutional review

The study was approved by the institutional review board of Charité—Universitätsmedizin Berlin (approval number EA2/074/24). In accordance with the retrospective design of this study, the requirement for written informed consent was waived by the institutional review board.

### Study sample and design

This single-center retrospective feasibility study included 30 pediatric patients with histologically confirmed NB who underwent clinically indicated MRI for initial imaging of the therapy-naïve tumor between June 2016 and September 2024. MRI of the primary tumor (located in the abdomen, thorax, pelvis, or neck) was analyzed. In patients with multiple lesions, only the primary tumor was included in the analysis. Patients were stratified into three risk groups (high-risk: n = 13; intermediate-risk: n = 4; low-risk: n = 13) according to standard clinical criteria. For binary classification, intermediate- and low-risk groups were pooled (n = 17 vs. high-risk: n = 13), given substantially different treatment intensity and prognosis. The MRI protocol comprised DWI, T2w, and postcontrast T1w (0.1 mmol/kg gadobutrol, Gadovist®, Bayer Pharma AG; no precontrast T1w was acquired), consistent with standard clinical practice. Patients were excluded for prior tumor-directed therapy, unavailable sequences, or non-diagnostic image quality. MRI was performed under anesthesia, sedation, or awake, per clinical need.

### MRI acquisition

All data were acquired on a single 3-Tesla MRI scanner (Siemens, Magnetom Skyra). Imaging was conducted according to standardized clinical protocols, with minor parameter adjustments (e.g., slice coverage, field of view) to ensure complete tumor inclusion and optimal image quality in the respective anatomical regions. Key acquisition parameters for the T1w, T2w, and DWI sequences (from which ADC maps were derived and used for feature extraction; raw DWI images were not used) are summarized in Table 1.

**Table 1:** MRI acquisition parameters.

Sequence	TR	TE	Slices	Pixel spacing	Slice thickness	b-values	Repetitions	Acquisition time
	in ms	in ms	in #	in mm	in mm	in s/mm^2^	in #	in min
T1w	3.5–5.3	1.5–2.3	40–288	0.68–1.2	0.8–4.0	–	1–2	2:10–7:05
T2w	1200–4100	38–130	40–170	0.42–1.1	2.0–3.0	–	1–4	2:40–4:56
DWI	950–8800	50–65	26–224	1.2–1.5	3.0–5.0	50, 500, 1000 or 50, 400, 800	1–20	2:20–7:26

Abbreviations: T1w, T1-weighted; T2w, T2-weighted; DWI, diffusion-weighted imaging.

### Preprocessing

Tumors were semi-automatically segmented using the built-in region-growing algorithm in 3D Slicer^20^ v5.6.2 and reviewed by a single radiologist with more than ten years of experience in pediatric oncology imaging. Segmentations were performed on T1w images and registered to T2w and ADC using elastix^21^ v4.9.0 with an identity transform and nearest-neighbor interpolation. Routine clinical data served as the reference standard for NB risk classification, based on the clinically assigned NB risk group determined by institutional multidisciplinary assessment using contemporary International Neuroblastoma Risk Group (INRG) based criteria^22^.

### Radiomics feature selection

Radiomic features were computed using MATLAB R2024a (MathWorks Inc., USA). Per sequence, 208 features were extracted following IBSI nomenclature, comprising 23 shape, 49 first-order intensity, and 136 texture features (GLCM, GLRLM, GLSZM, GLDM, NGTDM)^16^. This exceeds the 169 formally benchmarked features due to additional descriptors included in the MATLAB implementation. In multi-sequence analyses, shape features were extracted from a single sequence, as cross-sequence values differed only by negligible numerical precision.

Six feature selection strategies were employed, each using retention thresholds of 0.05, 0.1, 0.15, 0.2, and 0.25.

1. ANOVA-LASSO^23^: Features with *p* < retention threshold in one-way Analysis of Variance (ANOVA) were selected and then passed to Least Absolute Shrinkage and Selection Operator (LASSO) regression (α = 1), implemented with internal stratified tenfold cross-validation. Features with non-zero coefficients at the optimal model were retained.
2. ReliefF^24^: Features were ranked by their ability to discriminate between similar instances of different classes. Features were retained according to the retention threshold defining the percentage of top-ranked features selected.
3. Chi-square^25^: Features were ranked based on statistical dependence with class labels using chi-square scores. Features were retained according to the retention threshold defining the percentage of top-ranked features selected.
4. Tree-based Feature Importance^26^: Features were ranked by their contribution to decision splits in a trained Decision Tree classifier. Features were retained according to the retention threshold defining the percentage of top-ranked features selected.
5. Elastic Net^27^: Logistic regression with Elastic Net regularization (α = 0.5) and internal tenfold cross-validation. Features with non-zero coefficients were retained. The retention threshold determined the coefficient threshold for feature selection.
6. Minimum Redundancy Maximum Relevance^28^ (mRMR): Features were ranked by maximizing relevance to class labels while minimizing mutual redundancy. Features were retained according to the retention threshold defining the percentage of top-ranked features selected.

Feature selection was embedded within a LOO-CV loop. In each iteration, one subject was withheld, and all feature selection methods were applied to identify discriminative features from the remaining cohort. Feature occurrence frequencies were then aggregated across all iterations. Minimum selection frequency thresholds of 20%, 40%, 60%, and 80% were applied to retain features that were consistently selected across the leave-one-out iterations for subsequent model training.

### Machine learning classifier

Selected discriminative radiomics features were reduced in dimensionality using principal component analysis (PCA), retaining components that explained over 95% of the variance. These components served as input for classifier training to stratify tumor risk according to clinical risk classification as the reference standard. To mitigate overfitting in this small cohort and to reduce the multiple-comparisons burden, hyperparameters for each classifier were optimized within the inner LOO-CV loop using MATLAB’s default hyperparameter search space. Class-imbalance resampling was not applied due to the small sample size.

1. Support Vector Machine^29^ (SVM, Error-Correcting Output Codes framework, linear kernel)
2. Decision Tree^30^ (DT, Gini diversity index, minimum leaf size = 1)
3. K-Nearest Neighbors^31^ (K-NN, k = 5, Euclidean distance)
4. Random Forest^32^ (RF, bagging, random feature subsets at each split)
5. Extremely Randomized Trees^33^ (ERT, randomized feature thresholds at each node)
6. Extreme Gradient Boosting^34^ (XGB, maximum splits = 50)

### Performance evaluation

**Fig. 1:**
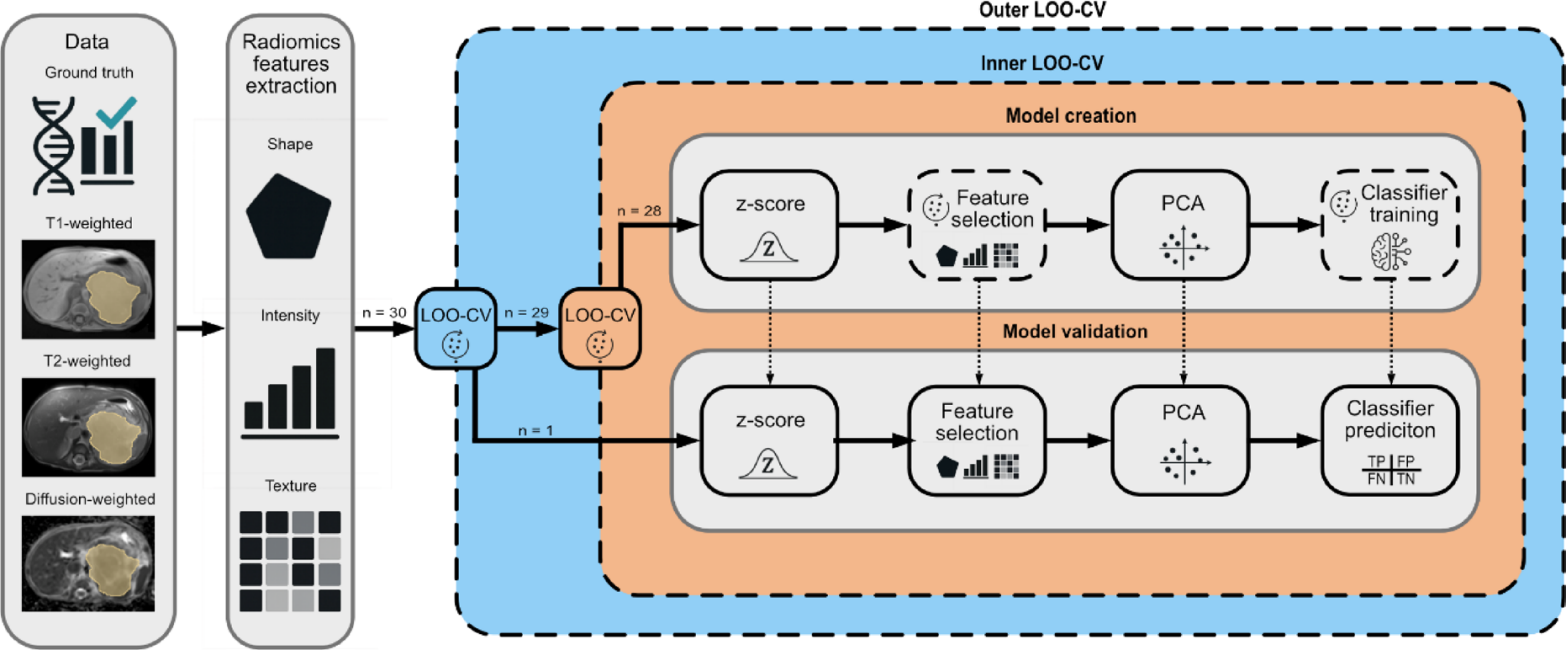
Overview of performance evaluation pipeline. The dataset (n = 30) includes T1-, T2-, and diffusion-weighted (from which ADC maps were derived and used for feature extraction) MRI volumes, tumor segmentations, and ground-truth risk labels. Radiomics features representing shape, intensity, and texture were extracted. Model performance was evaluated using a nested LOO-CV framework: the outer loop (blue) excluded one subject from all model development stages. Within each iteration of the inner loop (orange), one additional subject was withheld to generate model-prediction instances using z-score normalization, feature selection with internal LOO-CV, PCA, and classifier training. Within each outer fold, the inner models generated predictions for the inner held-out patients, so that each patient received n-1 cross-validated predictions from models trained on subsets that excluded that patient. These were averaged into a single patient-level prediction, yielding n patient-level scores used for performance estimation. Abbreviations: LOO-CV, leave-one-out cross-validation; PCA, principal component analysis.

Figure 1 shows the performance evaluation pipeline. Performance was assessed using a nested LOO-CV framework^35^, with reporting guided by the CLAIM checklist^36^. In each iteration (n_outer_ = n_all_) of the outer LOO-CV loop, one patient was withheld. Within the nested inner LOO-CV loop, an additional patient was excluded (n_inner_ = n_outer_-1), and model development (including z-score normalization, feature selection performed using an internal LOO-CV loop, PCA, and classifier training) was based on the remaining n_inner_ patients.

In each inner iteration, the resulting model generated a prediction for the patient held out within that inner fold, so that each patient received n-1 cross-validated predictions from models trained on subsets that excluded that patient. For each patient, these n-1 predictions were averaged into a single patient-level score, and the hard class assignment was obtained as the argmax of the two aggregated class scores (appropriate for SVM and XGB, whose raw outputs are decision margins rather than probabilities). Classification results were compared to clinical risk classification using confusion matrix analysis, and all six metrics, comprising the area under the receiver operating characteristic curve (AUC), accuracy, sensitivity, specificity, precision, and F1 score, were computed from these n patient-level observations. Because each patient-level prediction is the average of n-1 model instances, each reported result reflects an ensemble of models of the given type rather than a single trained classifier. Ninety-five percent confidence intervals were derived by stratified bootstrap (B = 2000 resamples of 13 high-risk and 17 low-/intermediate risk patients, percentile method, fixed seed), and the reported standard deviation is the corresponding bootstrap standard deviation^37^.

Pairwise AUC differences between classifiers were evaluated using the DeLong test^38^. To account for multiple comparisons, false discovery rate correction was applied using the Benjamini–Hochberg method^39^ across all seven MRI sequence configurations, covering all $\left(\genfrac{}{}{0pt}{}{6}{2}\right)=15$ classifier pairs per configuration (105 comparisons in total). Adjusted p-values were computed and evaluated against a threshold of 0.05. As a complementary, selection-free comparison, the proportion of candidate configurations per classifier that cleared the AUC > 0.7 threshold (the pass-rate) was also computed.

## Results

**Fig. 2:**
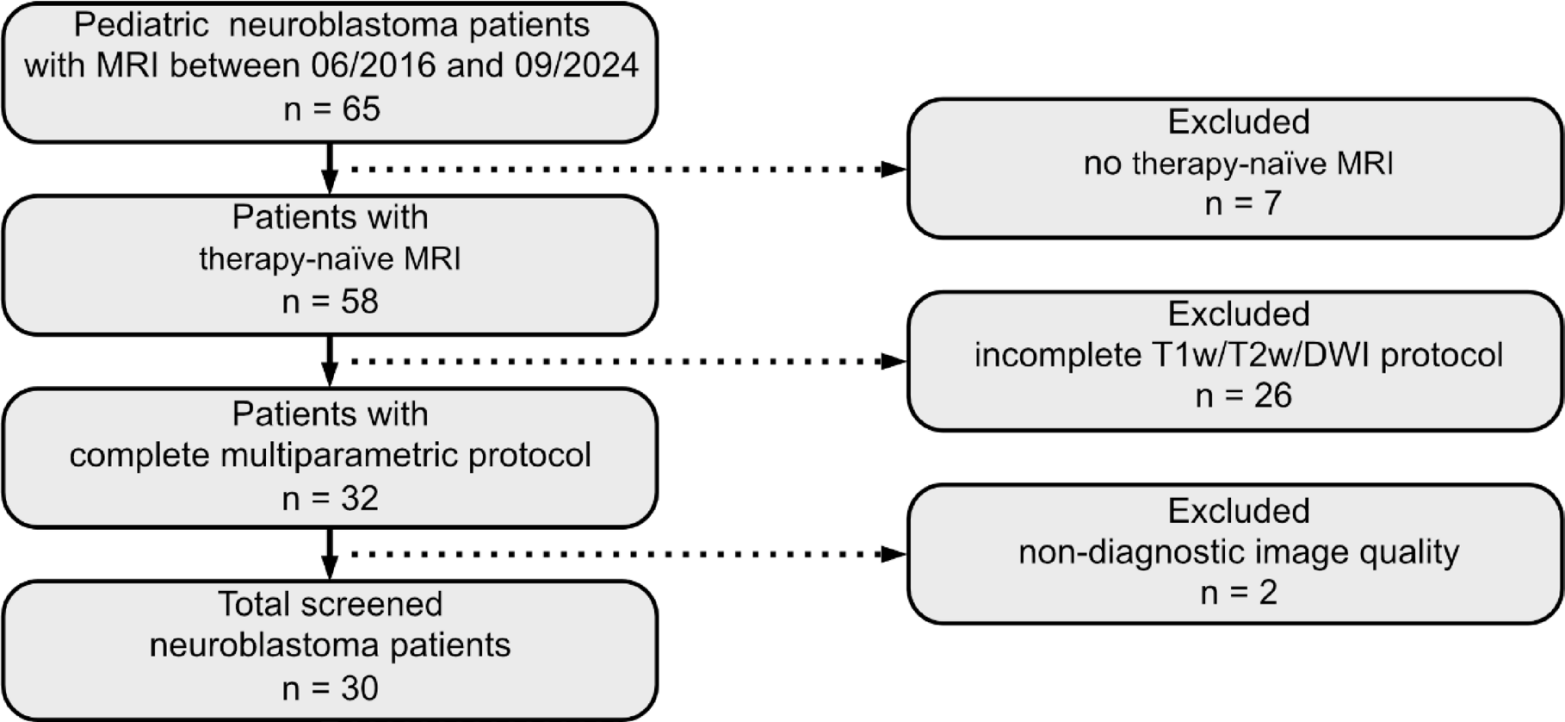
Flow chart illustrates selection process for the pediatric neuroblastoma study cohort. Abbreviations: T1w, T1-weighted; T2w, T2-weighted; DWI, diffusion-weighted imaging.

A total of 30 out of 68 pediatric patients (mean age ± standard deviation, 38 ± 40 months; age range, 1–187 months; 17 males) met the screening criteria (Fig. 2).

A total of 5040 combinations from feature selection through final prediction were evaluated. These combinations originated from the seven MRI sequence configurations (T1w, T2w, ADC, T1w + T2w, T1w + ADC, T2w + ADC, T1w + T2w + ADC). For each sequence configuration, six feature selection methods were applied (ANOVA-LASSO, ReliefF, chi-square, Tree-based Feature Importance, Elastic Net, and mRMR) with five feature retention thresholds (0.05, 0.1, 0.15, 0.2, 0.25). Each method was evaluated across four selection frequency thresholds (20%, 40%, 60%, 80%) within the LOO-CV feature selection loop, followed by training six ML classifiers (SVM, DT, K-NN, RF, ERT, and XGB). Among all evaluated combinations, 826 (16.39%) achieved an AUC > 0.7.

Among models with AUC > 0.7, chi-square feature selection was most prevalent (30.8%), followed by ReliefF (21.4%), mRMR (19.5%), Tree-based Feature Importance (17.9%), Elastic Net (5.9%), and ANOVA-LASSO (4.5%). Higher selection-frequency thresholds were somewhat more frequent among high-AUC models, with 80% contributing to 29.2% of high-AUC models. Stricter thresholds consistently retained fewer features, with medians of 14, 22, 29, and 39 at the 80%, 60%, 40%, and 20% thresholds, respectively. Across folds and sequence configurations, the number of features retained after frequency-based selection had a median of 26 (range 8–54), prior to PCA, which retained a median of 7 components (range 1–17).

**Fig. 3:**
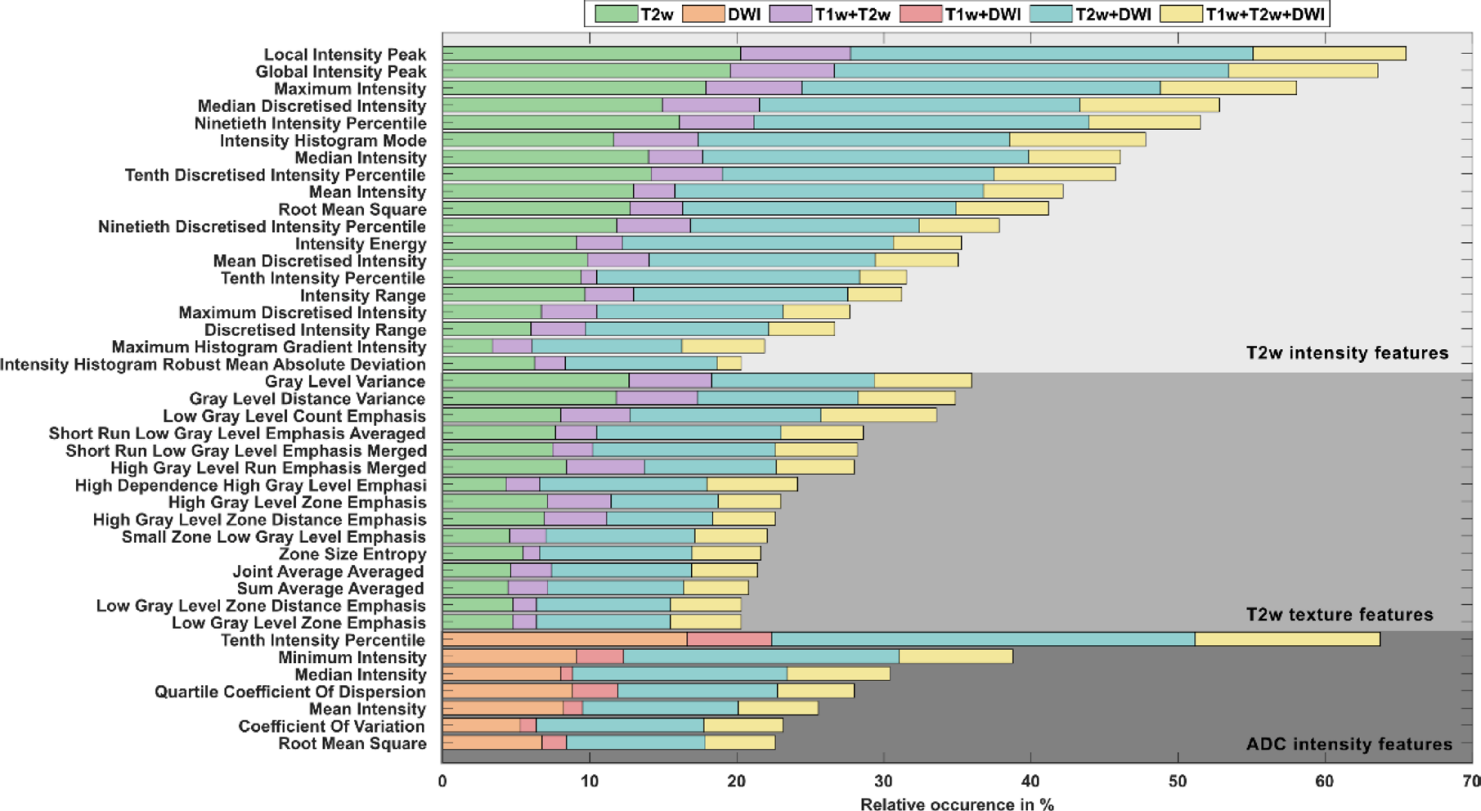
Most frequently selected radiomics features across MRI sequences contributing to accurate neuroblastoma risk stratification. Bars indicate the normalized frequency of feature selection across nested cross-validation folds where model performance exceeded AUC > 0.7. Only features selected in more than 20% of cases are shown. Grey-shaded background bands group features by sequence and type: T2w intensity (light grey), T2w texture (medium grey), and ADC intensity (dark grey). Texture and intensity features from T2w, and intensity features from DWI, were most prevalent in predictive performance. Neither T1w nor shape features were selected in more than 20% of cases and were therefore not displayed. Feature selection was performed using ReliefF, chi-square, Tree-Based Feature Importance, Elastic Net, mRMR, and ANOVA-LASSO algorithms within a LOO-CV framework. Abbreviations: T1w, T1-weighted; T2w, T2-weighted; ADC, apparent diffusion coefficient; mRMR, Minimum Redundancy Maximum Relevance; ANOVA-LASSO, Analysis of Variance—Least Absolute Shrinkage and Selection Operator; LOO-CV, leave-one-out cross-validation.

Figure 3 shows that the most frequently selected features were dominated by intensity descriptors (only those exceeding the 20% minimum selection threshold are displayed), with fewer texture features and no shape features among the top 41. T2w contributed both intensity and texture features, whereas ADC features were exclusively intensity-based, supporting the central role of T2w and ADC in the best-performing models.

Features from T2w and ADC were the main contributors to models achieving AUC > 0.7, with T2w + ADC yielding the largest number of successful configurations (261), followed by T2w alone (168) and ADC alone (140). By contrast, configurations involving T1w were far less common among high-performing models, indicating a minimal contribution of T1w-derived features to the best results.

The ten most frequently selected features were all intensity-based. Local Intensity Peak, Tenth Intensity Percentile, Global Intensity Peak, and Maximum Intensity exhibited the highest overall occurrences. Nine features were derived from the T2w sequence. One feature, Tenth Intensity Percentile, was from the ADC map.

**Fig. 4:**
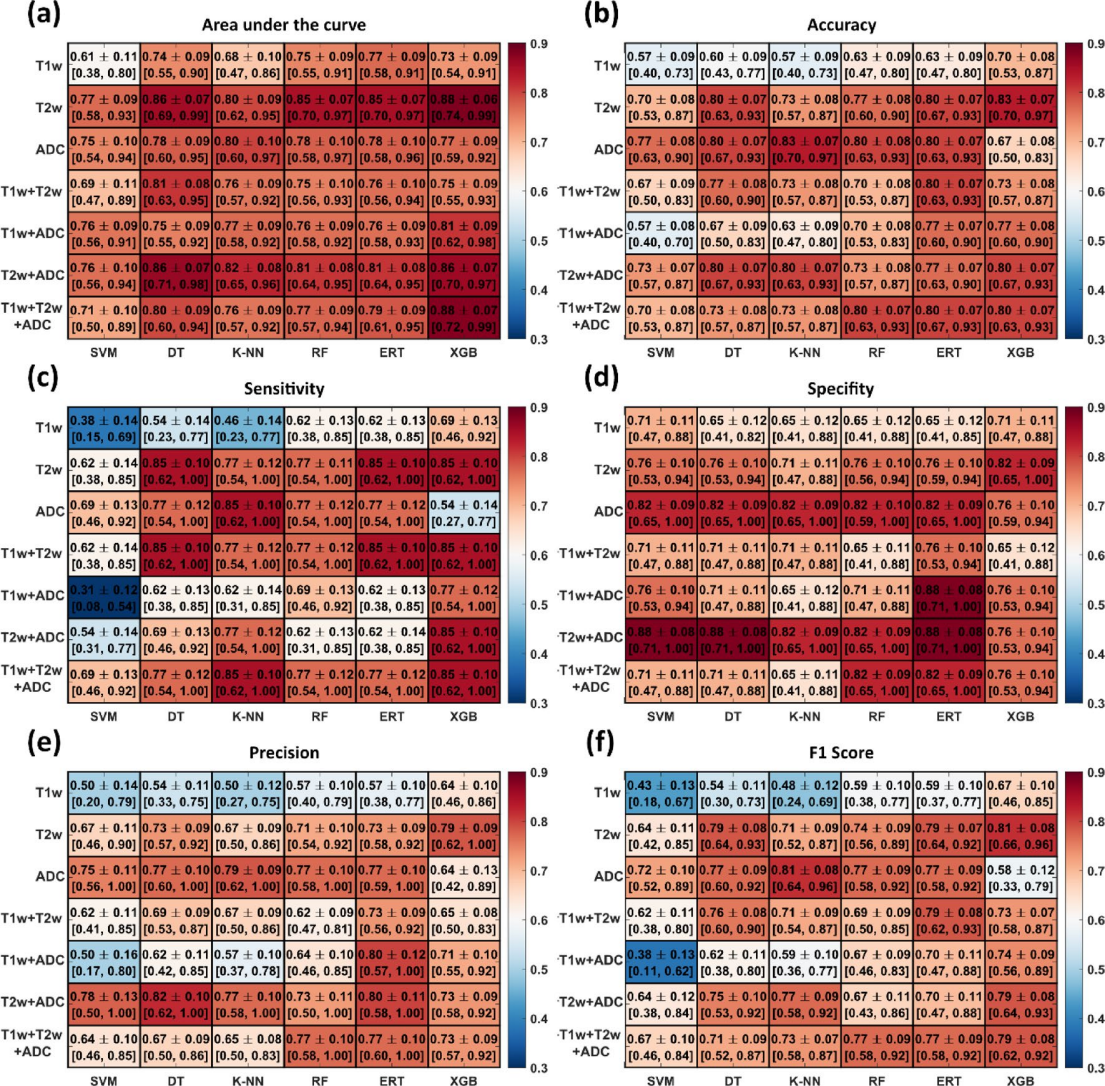
Heatmaps of evaluation metrics for machine learning classifier in radiomics based neuroblastoma risk stratification. Metrics include (**a**) AUC, (**b**) Accuracy, (**c**) Sensitivity, (**d**) Specificity, (**e**) Precision, and (**f**) F1 score. Rows correspond to MRI sequence combinations (T1w, T2w, ADC, T1w + T2w, T1w + ADC, T2w + ADC, T1w + T2w + ADC), and columns to classifiers (SVM, DT, K-NN, RF, ERT, XGB). Each cell shows the point estimate ± bootstrap standard deviation in the top row and the lower and upper bounds of 95% CI in the bottom row, obtained from the stratified bootstrap. Several cells reach comparable values with overlapping confidence intervals, so the maxima do not identify a single best model. Abbreviations: AUC, area under the curve; SVM, Support Vector Machine; DT, Decision Tree; K-NN, K-Nearest Neighbors; RF, Random Forest; ERT, Extremely Randomized Trees; XGB, Extreme Gradient Boosting; LOO-CV, leave-one-out cross-validation; T1w, T1-weighted; T2w, T2-weighted; ADC, apparent diffusion coefficient.

Radiomic model performance was quantified across the seven sequence combinations and six ML classifiers. Figure 4 reports metrics as the point estimate ± bootstrap standard deviation. The highest AUC (0.88) was observed for T2w and for the T1w + T2w + ADC combination, both with XGB (0.88 ± 0.06 and 0.88 ± 0.07, respectively), closely matched by several other T2w and T2w + ADC configurations (AUC 0.85–0.86). After correction for multiple comparisons, no pairwise difference between classifiers reached statistical significance, and the limited precision is consistent with the pilot sample of 30 patients. The Brier scores of the probability-output classifiers ranged from 0.15 to 0.19. Brier scores were not computed for SVM and XGB, whose outputs are decision margins rather than probabilities.

After correction for multiple comparisons, differences between classifiers did not reach significance, and these results do not permit conclusions about the relative performance of individual classifiers. SVM was nonetheless the weakest classifier, clearing AUC > 0.7 in only 2.5% of configurations compared with 18% to 21% for the other classifiers. This pass-rate reflects a separate, selection-free comparison across all candidate configurations per classifier, distinct from the DeLong tests. At the individual-metric level, the main trade-off was between sensitivity and specificity. The highest observed sensitivity (0.85) was reached by several classifiers (DT, ERT, K-NN, and XGB) across the T2w, ADC, and combined sequences, suggesting fewer high-risk cases would be missed, whereas the highest observed specificity (0.88) was reached by several classifiers (SVM, DT, and ERT) on the T1w + ADC and T2w + ADC sequences, suggesting fewer low/intermediate-risk cases would be overcalled as high risk. The highest accuracy (0.83) and F1 score (0.81) were each reached by two configurations (T2w with XGB and ADC with K-NN). Because several classifiers reached the same top values for each metric and no between-classifier difference reached significance, these maxima do not identify a single best model. The comparable performance of the T1w + T2w + ADC configuration most plausibly reflects its T2w and ADC components, given that no T1w feature exceeded the selection threshold.

## Discussion

This exploratory pilot study provides preliminary evidence that multiparametric MRI radiomics may support risk stratification in pediatric NB. The highest overall discrimination (AUC 0.88) was obtained with T2w features and with the combined T1w + T2w + ADC features, closely followed by several other T2w and T2w + ADC configurations. Corresponding Brier scores (0.15–0.19) for the probability-output classifiers were relatively low, suggesting reasonable probabilistic calibration. If maximizing sensitivity is the priority, the highest sensitivity (0.85) was reached by several classifiers rather than any single model. Feature selection consistently favored intensity and texture descriptors from T2w and ADC. This aligns with clinical understanding that these sequences capture critical tumor heterogeneity and microstructural properties relevant to NB risk stratification. Intensity and texture features consistently dominated across configurations, whereas shape descriptors were discarded during feature selection, suggesting that NB risk is driven more by internal heterogeneity than by gross morphology. The stronger contribution of T2w and ADC-derived features over T1w underlines the complementary value of these sequences, consistent with prior reports showing that diffusion metrics (e.g., apparent diffusion coefficient) reflect NB aggressiveness and relapse risk^40^.

Among feature selection methods, chi-square selection was the most frequently represented among models achieving AUC > 0.7 (30.8%), followed by ReliefF and mRMR. Higher minimum selection thresholds were more frequently represented among models achieving AUC > 0.7. Notably, the 80% threshold contributed 29.2% of all high-performing configurations, more than the 60% (26.5%), 40% (24.3%), and 20% (20.0%) thresholds. These findings suggest that features consistently selected across validation folds may be more stable in this dataset. Elastic Net contributed a smaller share of high-performing models (5.9%), which may reflect parameter-tuning sensitivity for this dataset.

The ten most frequently selected features were exclusively first-order intensity-based, dominated by T2w-derived metrics with one from ADC. T2w intensity peaks may reflect signal heterogeneity associated with necrosis or hemorrhage, though T2 signal lacks histopathologic specificity^41^. The low percentile ADC feature may reflect low diffusion tumor compartments, potentially associated with increased cellularity^42^. This biological interpretability aligns with pediatric studies linking T2w intensity heterogeneity to tumor grade and prognosis^43^.

While established risk stratification in NB relies on invasive clinicomolecular profiling, purely clinical or stagingbased prognostic models show only modest to moderate discrimination^44^. The INRG classification system^45^, which defines risk groups based on stage, age, histology, MYCN status, chromosomal aberrations and DNA ploidy, does not incorporate quantitative MRI, reflecting the substantial residual uncertainty of clinical risk assignment alone. Within this context, our imagingonly multiparametric MRI radiomics models may provide complementary prognostic information alongside established histopathologic and molecular risk markers.

Our findings align with and extend recent work in pediatric oncology radiomics. For example, Kim et al.^43^ applied radiomics to NB in 46 patients (32 training, 14 test) using a single axial slice of the largest tumor section on T2w MRI. They selected texture and wavelet features from that slice and built logistic regression and RF models achieving AUCs ≈ 0.94 in both training and test cohorts. In contrast, we used the whole-tumor volume across T2w and DWI sequences, capturing a greater extent of intratumoral heterogeneity than any 2D approach. Consistent with Kim et al., our analysis found T2w texture/intensity features to be highly predictive. T2-weighted and DWI sequences produced the largest number of configurations above AUC > 0.7 (261 vs 168 for T2w alone, which achieved the highest single AUC), reflecting a trade-off between peak discrimination and robustness, as expected given the established association of diffusion metrics with NB aggressiveness (e.g., lower apparent diffusion coefficient values with higher relapse risk). Across the classifiers evaluated, the highest AUC was 0.88; after correction for multiple comparisons no classifier significantly outperformed another. SVM was the weakest classifier in a selection-free comparison, clearing AUC > 0.7 in only 2.5% of configurations, while the highest sensitivity (0.85) was reached by several classifiers rather than any single model. Consistent with the pilot nature of the study, several classifiers reached similar performance at this sample size, and the data do not support ranking individual classifiers or attributing particular strengths, such as higher sensitivity, to specific model families.

Luo et al.^46^ extracted 1930 features across multiparametric brain MRI in 64 pediatric medulloblastoma patients and used SVM to achieve AUC = 0.84 for pediatric brain-tumor risk. Like prior work, our study shows that radiomic features, particularly first-order intensity descriptors, capture aspects of tumor heterogeneity that are not readily appreciable on visual inspection. In NB specifically, imaging-based biomarkers (beyond size and stage) have shown promise: a recent systematic review by Wang et al.^47^ found that radiomics based ML models detect high-risk molecular markers (e.g. MYCN amplification) with pooled AUC≈0.94. That review emphasizes the need for non-invasive predictors of risk (since biopsy can miss heterogeneity). Our work adds to this by providing a whole-tumor multiparametric MRI radiomics framework.

The employed nested LOO-CV framework follows best practices for small datasets as described by An et al.^48^. In addition to the outer and inner loops, additional LOO-CV steps were implemented within feature selection and classifier training to optimize model parameters, ensure robust identification of discriminative features, and prevent information leakage. This design maximized training data per fold while producing held-out predictions, thereby reducing, but not eliminating, optimistic bias.

This study has several limitations. The small cohort size, while reflecting the rarity of pediatric NB, limits statistical power; with only 30 patients, no pairwise comparison between classifiers reached significance after correction, so the findings support feasibility rather than definitive clinical implementation. Because intermediate-risk tumors can biologically resemble either low- or high-risk disease, pooling them with low-risk patients may obscure heterogeneity and complicate performance interpretation. The retrospective single-center design introduces selection bias, and the single-scanner protocol limits reproducibility across vendors, field strengths, and acquisition settings. Segmentation reproducibility was not formally assessed. The eight-year acquisition period may have introduced additional temporal protocol variability, including variability in voxel size and contrast parameters. Although a rigorous nested LOO-CV framework was implemented, these validation schemes have recently been shown to be susceptible to distributional bias^35^, making external validation on independent datasets essential. Each reported result reflects the average of 29 model instances rather than a single trained classifier. In addition, the best-performing configuration for each sequence and classifier was chosen by maximising AUC across many candidate configurations evaluated on the same patients, so the reported performance is likely optimistic relative to a configuration specified in advance. Percentile bootstrap intervals at n = 30 have only approximate coverage, and because several metrics are discrete, the intervals are granular and several reach the boundary. Prospective multi-institutional studies with harmonized imaging protocols and repeated segmentations are needed. Finally, integrating genomic, molecular, and histopathologic data may improve prediction and better capture NB risk.

Despite these limitations, this study supports the feasibility of multiparametric MRI radiomics for non-invasive risk stratification of pediatric NB. Whole-tumor, 3D radiomic extraction and the integration of T2w and DWI sequences enabled characterization of intratumoral heterogeneity beyond that of single-modality approaches; that said, a single sequence achieved the highest individual AUC, while the combination produced the larger number of high-performing configurations, reflecting a trade-off between peak discrimination and robustness. Intensity and texture features from T2w and ADC emerged as the dominant contributors to model performance. Although contrast-enhanced T1w features contributed little to the models, tumor delineation relied on postcontrast T1w imaging and no non-contrast protocol was evaluated; these data therefore do not support omitting gadolinium administration in this population. Future work should focus on external validation in multi-institutional cohorts and on integrating radiomics with clinical and molecular variables to assess incremental prognostic value. If confirmed, MRI-based radiomic profiling could ultimately support non-invasive, individualized risk assessment in children with NB.

## Conclusion

In summary, this study supports the potential of multiparametric MRI radiomics as a noninvasive imaging biomarker for pediatric NB risk stratification. By capturing intratumoral heterogeneity from whole-tumor MRI volumes, radiomics signatures offer complementary information to established clinical, genetic, and histologic markers. Further studies in larger, multi-institutional cohorts are needed to validate these findings and assess whether radiomics-based models could ultimately contribute to earlier complementary pretreatment risk assessment and inform more individualized treatment strategies for children with NB.

## Data Availability

The raw imaging data are not publicly available due to patient privacy regulations. The anonymized radiomic feature matrices extracted in this study are available from the corresponding author upon reasonable request.
